# Microbial community dynamics in the rhizosphere of a cadmium hyper-accumulator

**DOI:** 10.1038/srep36067

**Published:** 2016-11-02

**Authors:** J. L. Wood, C. Zhang, E. R. Mathews, C. Tang, A. E. Franks

**Affiliations:** 1 Department of Microbiology, La Trobe University, Melbourne Campus, Victoria, 3086, Australia; 2 Center for AgriBiosciences, La Trobe University, Melbourne Campus, Victoria, 3086, Australia

## Abstract

Phytoextraction is influenced by the indigenous soil microbial communities during the remediation of heavy metal contaminated soils. Soil microbial communities can affect plant growth, metal availability and the performance of phytoextraction-assisting inocula. Understanding the basic ecology of indigenous soil communities associated with the phytoextraction process, including the interplay between selective pressures upon the communities, is an important step towards phytoextraction optimization. This study investigated the impact of cadmium (Cd), and the presence of a Cd-accumulating plant, *Carpobrotus rossii* (Haw.) Schwantes, on the structure of soil-bacterial and fungal communities using automated ribosomal intergenic spacer analysis (ARISA) and quantitative PCR (qPCR). Whilst Cd had no detectable influence upon fungal communities, bacterial communities underwent significant structural changes with no reduction in 16S rRNA copy number. The presence of *C. rossii* influenced the structure of all communities and increased ITS copy number. Suites of operational taxonomic units (OTUs) changed in abundance in response to either Cd or *C. rossii*, however we found little evidence to suggest that the two selective pressures were acting synergistically. The Cd-induced turnover in bacterial OTUs suggests that Cd alters competition dynamics within the community. Further work to understand how competition is altered could provide a deeper understanding of the microbiome-plant-environment and aid phytoextraction optimization.

Heavy metals are problematic contaminants due to their toxicity, persistence in the environment, ability to enter the biosphere, and capacity to accumulate up the tropic levels of the food web^1^,^2^. Plant-based remediation strategies, such as phytoextraction, can facilitate the *in situ* removal of heavy metals from soils and water, and are generally considered environmentally friendly, cost-effective methods for precluding heavy metals from entering the biosphere^3^,^4^.

Multiple studies have provided evidence that soil microorganisms can improve the accumulation of heavy metals in plants and thus improve phytoextraction^5^,^6^,^7^,^8^,^9^,^10^. Microorganisms used to augment phytoextraction are typically isolated from heavy-metal-contaminated soils and rhizospheres (the root-soil interface)^6^,^11^,^12^,^13^,^14^. Increases in plant tissue-concentrations of nickel, cadmium, zinc and selenium as high as 38%, 57%, 91% and 103%, respectively, have been reported following inoculation with microorganisms^5^,^6^,^8^,^9^,^10^,^15^.

Research investigating the impact of a single microbial inoculum on specific plant species is important for the development of efficient phytoextraction but potentially overlooks the complexity of the plant-soil environment. Indigenous soil- and rhizosphere-microbial communities are a significant *in situ* variable with the potential to inhibit or out-compete phytoextraction-assisting inocula^16^. The importance of indigenous microbial communities in metal accumulation was highlighted by microcosm studies which reported that disturbance (gamma-irradiation) of native soil communities significantly reduced Cd- and Zn-accumulation by *Arabidopsis halleri,* even though plant biomass was unaffected^17^.

Investigations that seek to understand changes to the *in situ* microbial community during phytoextraction could provide a deeper understanding of the microbiome-plant-environment^18^. For instance, studies that tracked the rhizosphere communities of *Sedum plumbizincicola* in Cd contaminated soil found that the indigenous community was significantly altered following inoculation with a single plant-growth-promoting bacterium^13^. The changes to the community, which remained even when the initial inoculum was no longer detectable, included decreased diversity and the enrichment of indigenous plant-growth-promoting (PGP)-rhizosphere bacteria. This research raises questions regarding the importance of indigenous bacteria in improving phytoextraction via PGP^13^. Addressing such questions requires an understanding of the ecology of rhizosphere communities associated with the phytoextraction process.

Rhizospheres are considered to be nutrient rich environments for microorganisms due to additional C and N inputs in the form of plant root exudate^19^. Root exudates can govern the assembly of the rhizosphere community and can select for rhizosphere microorganisms in both a positive and negative manner^19^,^20^,^21^,^22^,^23^. Specialized carbon compounds in root exudates select for microorganisms that specialize in their degradation, whilst antimicrobial compounds, such as phytoalexins, remove microorganisms from the community^24^. Thus, as an ecological niche the rhizosphere is characterized by plant-based selection and competition for space and nutrients.

Heavy metals present in contaminated soils targeted for phytoextraction also alter the structure of soil microbial communities. Heavy metals have been documented to increase the prevalence of heavy metal-resistant bacteria and are thought to increase the rate of horizontal gene transfer of genetic elements such as those encoding heavy metal resistance^25^,^26^ Cd toxicity has been reported to alter soil-microbial-community structure, reduce total microbial carbon and nitrogen, and increase the microbial C:N ratio^27^,^28^. These effects are attributed to Cd toxicity inducing cellular survival mechanisms that divert C from growth-related processes, reducing the amount of C incorporated into microbial biomass^28^. Observations that Cd toxicity also reduce microbial carbon-use efficiency corroborate this hypothesis^29^.

This work aimed to examine the interplay between selective pressures imposed by plants and soil contaminants on the structure of indigenous soil communities. The heavy metal, Cd, and a recently identified Cd-accumulating plant, *Carprobrotus rossii,* were used as selective agents to examine bacterial and fungal community structure through community fingerprinting and qPCR. We hypothesized that the presence of multiple selective pressures would create a narrow ecological niche that would select for unique suites of operational taxonomic units (OTUs).

## Results

Despite the high cadmium (Cd) doses used in this experiment, we observed no significant change in the biomass (fresh weight) of plant shoots (see Supplementary Fig. S1). Similarly, throughout the eight-week experiment Cd-treated plants did not exhibit any symptoms of Cd toxicity such as wilting or chlorosis (see Supplementary Fig. S1).

### Effect of selective pressures on total fungi and bacteria

**Figure 1:**
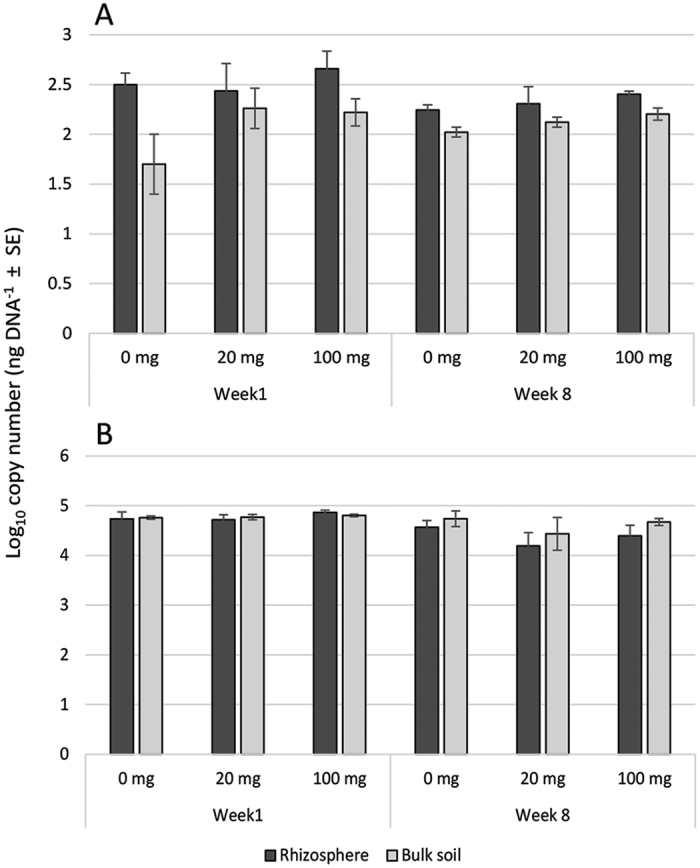
(**A**) Quantitative PCR estimation of fungal gDNA ITS copy number across treatments and sampling times. Two-way ANOVAs showed a significant difference in ITS copy number between bulk and rhizosphere communities in both weeks 1 (F_(1,24)_ = 9.23, p < 0.01) and 8 (F_(1,24)_ = 11.21, p < 0.01). (**B)** Quantitative PCR estimation of bacterial gDNA 16S rRNA copy number across Cd-treatments and sampling times showed no significant difference. Values are the reported as mean of n = 5 biological replicates ± SE.

Irrespective of Cd treatments, there was a significant difference in the copy number of the fungal internal transcribed spacer (ITS) between bulk and rhizosphere communities in weeks 1 (F_(1,24)_ = 7.36, p < 0.05) and 8 (F_(1,24)_ = 8.39, p < 0.01) as determined by factorial ANOVAs of qPCR data (Fig. 1A). In contrast, neither Cd-treatments nor the presence of the plant affected the copy number of bacterial 16S rRNA (Fig. 1B).

### Effect of selective pressures on microbial community structure

After removing singletons, ARISA-PCR identified a total of 167 unique bacterial and 48 unique fungal OTUs across all samples.

**Figure 2:**
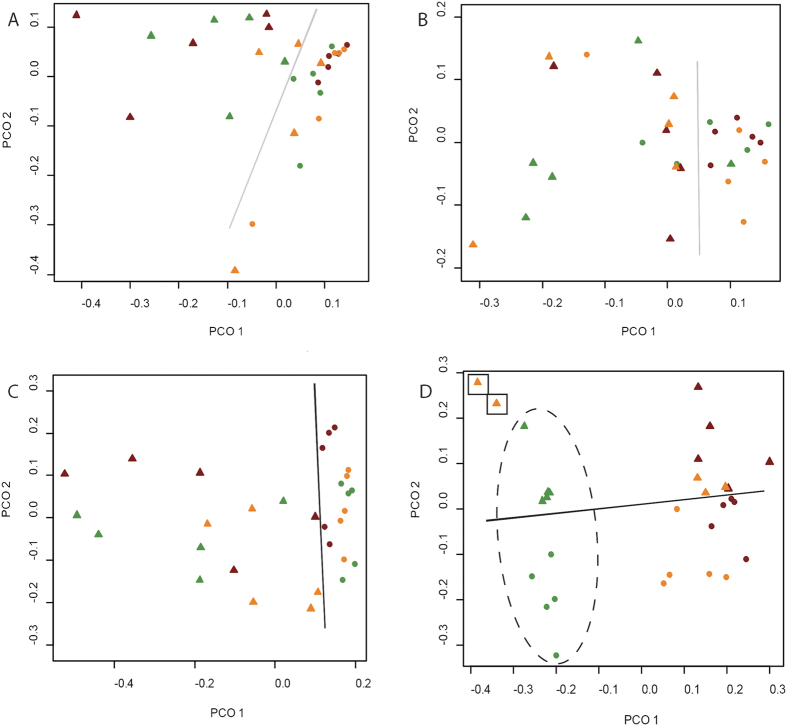
PCoA ordination of fungal (**A,B**) and bacterial (**C,D**) communities of bulk (•) and rhizosphere (Δ) soils sampled in weeks 1 (**A–C**) and 8 (**B–D**). Colour is indicative of Cd treatment: Green, 0 mg Cd kg soil ^−1^; Amber, 20 mg Cd kg soil ^−1^; Red, 100 mg Cd kg soil ^−1^. Solid line showing rhizosphere effect; dashed circle showing Cd effect; boxed data points are outliers.

Moderate grouping of fungal and bacterial communities due to the presence or absence of the rhizosphere (rhizosphere_pa_) was observed in weeks 1 and 8 using principal coordinates analysis (PCoA) (Fig. 2). In general, bulk soil communities formed tighter clusters than rhizosphere communities. Global R statistics for rhizosphere_pa_ comparisons were higher for bacterial communities in both weeks 1 and 8 compared to fungal communities (Table 1).

Minor but significant grouping of fungal communities due to Cd-treatments was detectable in week 1 but was lost by week 8 (Table 1). Conversely, minor grouping of bacterial communities in week 1, due to Cd-treatment, developed into a strong, significant community groupings by week 8 (R = 0.6; Table 1). The global R statistic for week 8 Cd-treatments was due to large, significant (after Bonferroni corrections; *p* ≤ 0.003) grouping between the 0 and 20 mg kg soil^−1^ (R_0–20_ = 0.64), and 0 and 100 mg kg soil^−1^ (R_0–100_ = 0.99) Cd-treatments.

**Figure 3:**
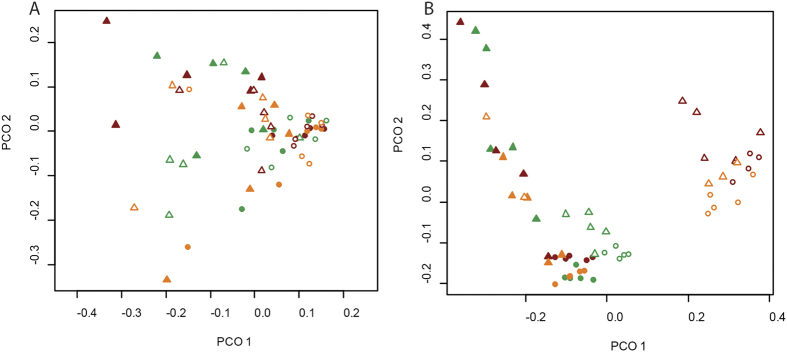
PCoA ordination of fungal (**A**) and bacterial (**B**) communities of the rhizosphere (Δ) and bulk (•) soils sampled in weeks 1 (solid symbols) and 8 (open symbols). Colour is indicative of Cd treatment: Green = 0 mg Cd kg soil ^−1^; Amber = 20 mg Cd kg soil ^−1^; Red = 100 mg Cd kg soil ^−1^.

Fungal communities were similar between weeks 1 and 8. However, bacterial communities in both rhizosphere and bulk soils changed significantly over time (R_week_ = 0.47, p < 0.05) (Fig. 3). Whilst in uncontaminated soils, the communities from week 8 grouped closely to those in week 1 in 2-dimensions, with the exception of two replicates week 8 bacterial communities at 20 and 100 mg Cd kg soil ^−1^ were distinct from all other communities irrespective of treatment or time (Fig. 3B).

### Response of OTU abundances to Cd and *C. rossii* rhizosphere

There was a significant Cd dose x rhizosphere interaction on the Shannon diversity (F_(2,24)_ = 3.7, p < 0.05), and OTU richness (F_(2,24)_ = 3.6, p < 0.05) of fungal communities at week 8, whilst species evenness was unaffected by either Cd dose or rhizosphere_pa_ (Table 2). Bacterial community diversity metrics were unaffected by rhizosphere_pa_ but Cd decreased Shannon-Wiener diversity (F_(2,24)_ = 22.6, p < 0.05), community evenness (F_(2,24)_ = 22.8, p < 0.05) and OTU richness (F_(2,24)_ = 29.5, p < 0.05; Table 3).

**Figure 4:**
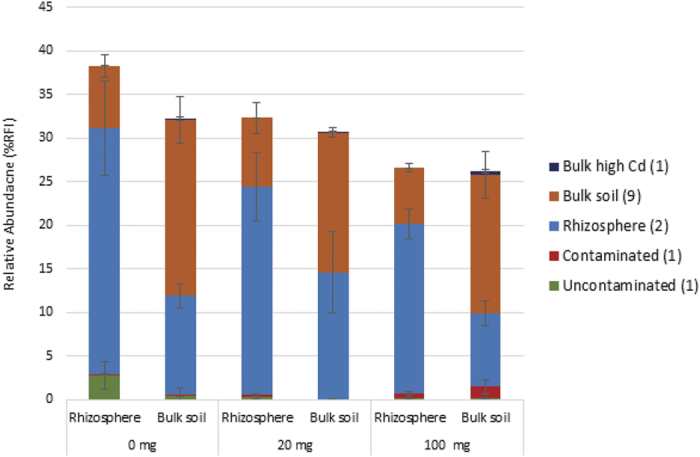
Relative abundance of total fungal OTUs with significant species indicator values across communities in soils treated for 8 weeks with 0, 20 and 100 mg Cd kg soil^−1^. OTUs are grouped based on the treatments or groups of treatments they were significantly (p < 0.05) enriched in. The number of OTUs contributing to each category is denoted in parenthesis in the key. Relative abundance values are the mean of n ± 5 biological replicates ± SE.

**Figure 5:**
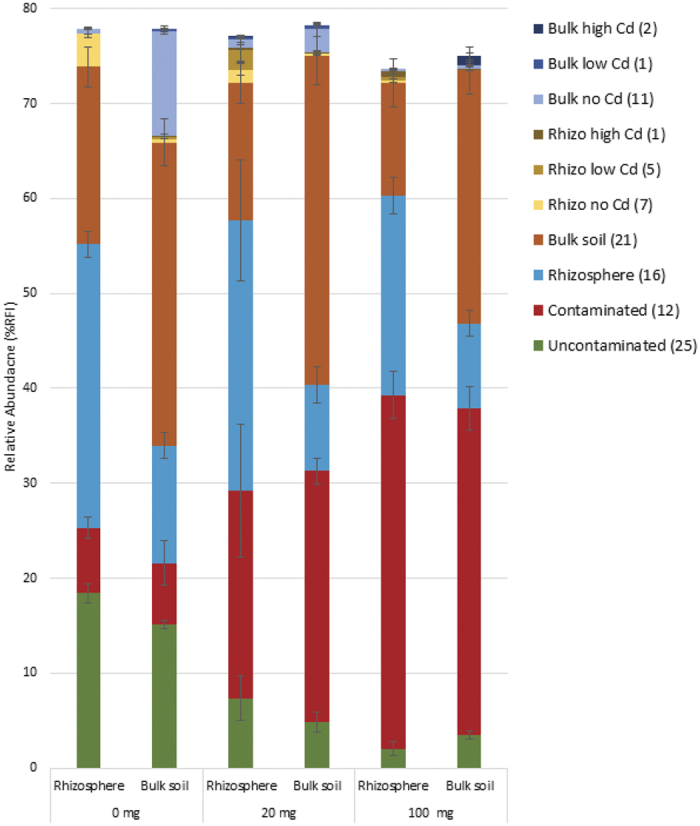
Relative abundance of total bacterial OTUs with significant species indicator values across communities in soils treated for 8 weeks with 0, 20 and 100 mg Cd kg soil^−1^. OTUs are grouped based on the treatments or groups of treatments they were significantly (p < 0.05) enriched in. The number of OTUs contributing to each category is denoted in parenthesis in the key. Relative abundance values are the mean of n = 5 biological replicates ± SE.

Species indicator tests identified 14 fungal and 101 bacterial OTUs that were significantly enriched (p < 0.05) in one of ten combinations of treatment groups (Figs 4 and 5). Fungal OTUs with significant IndVal scores were mostly enriched in the bulk or rhizosphere soils and collectively accounted for 25–35% of the average relative abundance for each community (Fig. 4). Bacterial communities contained OTUs that were enriched in the presence and absence of Cd as well as OTUs that were enriched in bulk and rhizosphere soils. Collectively these four groups of OTUs accounted for between 65% and 75% of the average relative abundance for each community. Most enriched OTUs were not unique to the groups of treatments. Bacterial communities in the uncontaminated soils had the highest number of unique OTUs (5). For any given group of treatments, the combined relative abundance of unique OTUs was less than 1.3%.

## Discussion

This study used automated ribosomal intergenic spacer analysis (ARISA) to identify the principal trends in soil microbial community structure in response to two selective pressures: Cd contamination and the rhizosphere. ARISA is a rapid, effective analysis technique that enables the structure of complex microbial communities, such as those associated with the rhizosphere, to be compared without the need for deep sequencing^30^,^31^. By monitoring the structural response of fungal and bacterial communities to increasing levels of Cd and the presence of a plant rhizosphere, we were able to detect different trends in the response of prokaryotic and eukaryotic proportions of the community to each selective pressure.

After eight weeks, fungal communities exhibited no significant grouping due to Cd, and species indicator tests only identified two OTUs that were enriched due to the presence or absence of Cd (Fig. 5). In contrast, ANOSIMs detected large (R = 0.6), significant grouping of bacterial communities in response to Cd at week 8 (Fig. 2). The Cd-induced changes to bacterial community structure were largely due to the turnover of OTUs enriched in Cd treatments rather than to changes in bacterial copy-number which is related to bacterial total biomass (Figs 1 and 5). Numerous studies have reported that Cd contamination reduces total microbial biomass carbon and as such we expected to observe a reduction in bacterial copy-number due to increasing Cd dose^27^,^28^. However, there have been reports whereby Cd load did not affect microbial biomass^32^.

The different responses of fungal and bacterial communities to heavy metals observed in this study have also been reported in the literature for over a decade. Different responses of prokaryote and eukaryote communities to heavy metals were first reported by Rajapaksha and colleagues using thymidine and acetate-in-ergosterol incorporation to measure bacterial and fungal activity, respectively^33^. Following Zn or Cu application, bacterial activity was reduced and then recovered whilst fungal activity spiked and then declined^33^. The authors proposed that the reduction and recovery of bacterial activity was likely to be due to an initial decline in community members poorly adapted to heavy-metal stress followed by the proliferation of remaining community members into the niches that were left vacant, causing a change in community structure. In addition to altering community structure, the proliferation of Cd-adapted OTUs into vacant niches would maintain the initial community biomass. Thus, this hypothesis may explain how Cd caused a turnover in bacterial OTUs without affecting 16S rRNA copy number, which was our observation in this study.

Unlike the bacterial communities, the structure of the fungal communities did not significantly change from week 1 to week 8, and initial grouping caused by Cd-treatments in week 1 were absent by week 8, suggesting that after an initial disturbance the community re-stabilized to its original state. Additionally, we did not observe any change in ITS copy number in response to Cd (Fig. 1). Our observation that fungal communities did not respond to Cd treatments is in accordance with reports from the literature which suggest that, compared to bacteria, fungi are less sensitive to a range of heavy metals including Cu, Zn and Cd^33^,^34^. The independent way in which prokaryotic and eukaryotic communities responded to Cd, indicates that the two communities were not significantly influencing each other but rather behaving as independent groups.

The influence of the rhizosphere on community beta diversity was detected using ordinations of fungal and bacterial communities and was present from week 1 (Fig. 2). Suites of OTUs enriched in the rhizosphere or bulk soils were detected for both fungal and bacterial communities (Figs 4 and 5). Rhizosphere-induced changes to fungal community structure were accompanied by increases in total ITS number, as detected by qPCR, and a trend towards reduced OTU richness in rhizosphere samples (Fig. 1, Table 2). These results are in accordance with Hilters original definition of the ‘rhizosphere’ as an environment with increased microbial density but reduced diversity due to selection by the plant^35^. The selective influence of the plant rhizosphere is well documented and has been repeatedly reported in the literature^21^,^36^,^37^. In contrast to the fungal community, rhizosphere-induced beta diversity changes to bacterial communities were not accompanied by changes to OTU richness or total 16S rRNA copy-number (Fig. 1, Table 3).

We hypothesized that where there was evidence of both selective pressures affecting community structure (i.e. in the bacterial community), the rhizosphere and Cd would act as multiple ecological filters under which few OTUs would be able to thrive. As such, we expected bacterial communities in the Cd-contaminated rhizosphere to exhibit a greater reduction in diversity, compared to bulk soils, or be occupied by a suite of unique OTUs. However, evidence of the two selective pressures having a combinatorial effect was minimal. We reported no change to bacterial copy-number or OTU richness in contaminated rhizospheres. Although rhizosphere specific OTUs were filtered from the community by Cd, only 4 OTUs were unique to contaminated rhizosphere communities. Collectively, these unique OTUs constituted less than 2% of the total relative abundance. Only one OTU was identified that had a significant positive correlation to both the rhizosphere and Cd dose. This suggests that although both the plant and the Cd shape the community, these selective pressures are largely acting on mutually exclusive suites of OTUs, rather than synergistically on the same OTUs. This suggestion is supported by the observation that the presence of the rhizosphere did not impact on the proliferation of Cd-resistant OTUs (Fig. 4). It is possible however, that OTUs unique to contaminated rhizosphere communities were present but were below the detection limit of ARISA^38^.

In this study, the Cd-induced changes in OTU abundance most likely reflected a change in what constitutes an adventitious growth strategy under high Cd contamination. We proposed that the OTUs responding positively to Cd exhibited some characters of Cd resistance, such as increased number of Cd-efflux pumps. Increases in heavy-metal-resistant community members in response to an increasing heavy-metal-selective pressure have been reported in the rhizosphere of *Alyssum mural,* a Ni hyper-accumulating plant^39^. The increase in heavy-metal-resistant microorganisms was attributed to increases in labile Ni caused by rhizosphere acidification. However, a pollution-induced community tolerance (PICT) analysis of the soil communities at week 8 from this study indicated that there was no increase in the Cd-resistant proportion of the community in response to Cd treatments (Fig. S2). Although PICT is confined to an analysis of the culturable proportion of the community, this data suggests that Cd resistance is not the key microbial function providing a selective advantage in the presence of Cd.

It is well documented that Cd can alter microbial community structure, but reports as to what types of microorganisms Cd selects for are conflicting in the literature. Hinojosa, *et al.*^40^ (2005) reported that heavy metal (Cd and Pb) contamination correlated positively with Gram-negative-associated fatty acids but negatively with fatty acids associated with actinomycetes and fungi. The authors reasoned that the filamentous nature of fungi and actinomycetes would increase their exposure to, and thus susceptibility to, environmental toxins such as heavy metals. Other authors using the same technique have reported increases in Gram positive bacteria in response to Cd^28^,^29^.

At the species level, previous studies have reported an enrichment of *Bacillus cereus* and *Enterobacter cloacae* in the presence of Cd^41^. However, the mechanism that facilitates their enrichment has yet to be identified. Ultimately, future work using transcriptomics or predictive functional profiling software, such as PICRUSt, is required to understand functional traits associated with the proliferation of specific OTUs in these conditions^42^.

### Conclusions and future perspectives

Ordinations and ANOSIMs of community fingerprinting data clearly indicate that prokaryotic and eukaryotic fractions of the soil microbial community responded independently to Cd contamination which had no detectable influence upon fungal communities but was the main driver of community structure for bacterial communities. Conversely, the influence of the rhizosphere upon community structure was subtle but present in both fungal and bacterial communities. Cd-induced changes elicited in bacterial communities were largely being driven by changes in the abundance of OTUs common to all treatments, indicating a shift in competition dynamics.

The increased relative abundance of OTUs in response to Cd was not affected by the presence of the rhizosphere, suggesting that selective pressures were not acting synergistically. Taxonomic and functional community profiling will be a logical and beneficial continuation of this investigation in order to elucidate functional traits enabling the proliferation of selected bacterial OTUs in a Cd-contaminated rhizosphere. Understanding the ecology of heavy-metal-contaminated soils will be integral for future phytoextraction work that requires the persistence of plant-growth-promoting isolates *in situ*.

## Materials and Methods

### Plant growth experiment

Plant growth experiments were conducted in glasshouses at La Trobe University, Bundoora, Victoria, Australia, using the Australian native succulent *Carpobrotus rossii*, which has been identified as a Cd-accumulator^4^.

Soil used in this experiment was a sandy loam collected from the topsoil (0–25 cm) of the La Trobe University Agricultural Reserve, Bundoora, Victoria, Australia. Prior to the experiment, soil was passed through a 2-mm sieve and air-dried. The soil had the following basic properties: 21.3% clay, 54.5% silt, 24.1% sand, 2.4% organic C, 2.75 g kg^−1^ total N, 0.076 dS m^−1^ electrical conductivity (1:5 water), pH 5.41 (1:5 soil:0.01 M CaCl_2_), 44 mg kg^−1^ Colwell P, 126 mg kg^−1^ Colwell K, 0.55 mg kg^−1^ Cd, and 119 mg kg^−1^ Zn.

#### Experimental setup

Soil (0.25 kg) was weighed into plastic bags and spiked with CdCl_2_ solution to achieve 20 mg kg^−1^ or 100 mg kg^−1^ Cd when required. The Cd doses chosen were reported to be below the critical value that causes 10% shoot biomass reduction (115 μg g^−1^)^4^. Distilled H_2_O was used as a negative control. Basal nutrients were added to each bag of soil, including those that would remain unplanted, to achieve the following concentrations (mg kg^−1^ soil): 150 K_2_SO_4_, 150 KH_2_PO_4_, 90CaCl_2_·2H_2_O, 21 MgSO_4_·7H_2_O, 14 MnCl_2_, 1.081 CuCl_2_·H_2_O, 10.33 ZnSO_4_·7H_2_O, 0.67 H_3_BO_3_ and 0.15 Na_2_MoO_4_·2H_2_O. Sterile distilled H_2_O was then added to wet the soil to 80% field capacity. Bags were allowed to settle for 2 h before being sealed and incubated at 25 °C for two weeks. Soils were mixed and rotated in the incubator every 1–2 days.

After two weeks of incubation, the soil was transferred to washed forestry tubes and *Carpobrotus rossii* plantlets were transplanted into half of the pots and the remaining half of the pots were left unplanted. Trays containing five replicates of each treatment (0, 20 & 100 mg Cd ± plants) were rotated in the glasshouse every two days for eight weeks to avoid spatial variation influences. To account for shading, replicates within trays were arranged in a randomized block design. Pots with and without plants were maintained at 80% field capacity, using sterile distilled H_2_O, for the duration of the experiment.

*Carpobrotus rossii* plants were propagated from cuttings in a heated sand bed for two weeks. Prior to transplanting, plantlets were removed from the sand and their roots were gently washed with distilled H_2_O, to remove propagation mix, before being transferred to the treatment pots.

#### Sampling procedure

Destructive sampling occurred at weeks one and eight of the experiment. Bulk soil samples were collected from replicates without plants by taking 5-cm cores of soil using 50 ml centrifuge tubes. Cores were stored at −80 °C until DNA extraction. Samples of rhizosphere soil were collected from replicates containing plants by gently removing the plants from their pots and shaking away the loose soil from the roots. The above-ground material was discarded and the roots, along with the soil clinging to them, were stored at −80 °C until DNA was extracted. At the time of DNA extraction, soil clinging to the roots was used for extracting DNA from rhizosphere-soil-microbial communities.

### Soil DNA extraction

Community gDNA was extracted from bulk and rhizosphere soils (0.25 g) using a power-soil DNA isolation kit (MOBIO) as per manufacturer’s instructions. DNA concentrations were recorded using an Implen P-class Nanophotometer (p-330). All samples were normalized to working concentrations of 5 ng μl^−1^ and stored at −20 °C until required.

### ARISA-PCR

Automated ribosomal intergenic spacer analysis (ARISA) is a community fingerprinting technique that uses fluorescently labelled primers to amplify the non-coding DNA fragments between conserved rRNA genes. Although community fingerprinting uses taxonomically ambiguous ‘OTUs’ and is limited to the detection of only the most abundant community members (between 1–10% of the total community OTUs), it remains a reliable, cost-effective method for identifying significant biological patterns and elucidating the environmental variables driving them^43^,^44^. The intergenic spacer (IGS) located between the 16S rRNA and 23S rRNA genes was targeted for bacteria, whilst the internal transcribed spacer (ITS), located between the 18S rRNA and 28S rRNA genes, was used to characterize fungal communities^45^,^46^. OTUs were assigned within communities based on amplicon length polymorphisms and OTU abundance was estimated via the relative fluorescence intensity associated with each OTU.

PCR amplifications were carried out in 20 μl reaction mixes using a TProfessional TRIO combi-thermocycler (Biomertra) for bacterial ARISA-PCR and a CFX Connect Real-Time PCR Detection System (BioRad) for fungal ARISA-PCR. Reaction mixtures contained: 2.5 U TopTaq DNA polymerase (Qiagen), 0.5 pM of dNTP mix, 0.5 pM of each appropriate primer, 3 pM of MgCl_2_ and 5.0 ng or 10 ng of community DNA for bacterial or fungal reactions respectively. Primer pairs for bacterial and fungal ARISA-PCR were *16S-1392F* - *23S-125R* (5′-GYACACACCGCCCGT; 5′-GGGTTBCCCCATTCRG) and *ITS1F* - *ITS4* (5′-CTTGGTCATTTAGAGGAAGTAA; 5′-TCCTCCGCTTATTGATATGC), respectively^47^,^48^,^49^. Primers *16S-1392F* and *ITS4* were labelled with 6-carboxyfluorescein (6-FAM) at the 5′ end.

Cycle settings used for bacterial PCR reactions were as follows: initial denaturation, 3 min at 94 °C; 33 cycles of 1 min denaturation at 94 °C, 1 min annealing at 52 °C, 1.5 min extension at 72 °C; and 6 min final extension at 72 °C^46^. Cycle settings used for fungal PCR reactions were as follows: initial denaturation, 2 min at 96 °C; 30 cycles of 1 min denaturation at 96 °C, 1 min annealing at 55 °C, 2 min extension at 72 °C; and 10 min final extension at 72 °C^50^.

For each PCR reaction, 10 μl of unpurified PCR product was submitted to the Australian Genome Research Facility (AGRF) for fragment separation analysis via capillary electrophoresis on an AB 3730 DNA analyser. The data were analysed using AB GeneMapper software (Applied Biosystems).

### OTU binning strategy and matrix preparation for ARISA fingerprints

ARISA data (fragment size, peak height and peak area) for all samples were obtained from AGRF. A cut-off of 50 fluorescence units was used to exclude background noise.

Sample × OTU matrices containing binned OTUs were created using R (version 3.2.2)^51^. Imprecision in OTU size calling increases with the length of DNA fragments. To account for this, optimal bin sizes were determined for three fragment size ranges (0–700, 701–999 and 1000–1200 bp) using the ‘automatic binning script’ created by Ramette (2009) in the R programming language^52^,^53^. Bin sizes of 4, 5 and 12 bp, and 5, 4 and 4 bp were determined to be optimal for bacterial and fungal datasets, respectively.

Optimal bin sizes were used to create three differently binned matrices for each dataset using the ‘interactive binning algorithm’, created by Ramette (2009). Binned matrices were combined to create one binned matrix with different bin sizes applied to different DNA fragment-length ranges. To avoid stochastically detected OTUs influencing community comparisons, singletons were removed^38^. The ‘automatic’ and ‘interactive’ binning scripts are available with their respective manuals and examples online (http://www.ecology-research.com)^54^.

### Quantitative PCR

Quantitative PCR (qPCR) was used to quantify total bacterial and fungal DNA copy number as an indicator of abundance. Primer pairs 1114f-1275r, which target the bacterial 16S rRNA gene^55^, and ITS1f-5.8S, which target the ITS region^56^, were used to detect bacterial and fungal communities respectively. qPCRs were run on a CFX Connect Real-Time PCR Detection System (BioRad). Each 5-μl reaction solution contained 0.25 pM of each forward and reverse primer, 2.5 μl of SsoAdvanced Universal SYBR^®^ Green Super Mix, sterile DNA-free water and 5 ng of community DNA. Thermocycling conditions were 20 s at 95 °C followed by 40 cycles of 95 °C for 3 s and 61.5 °C for 30 s for bacterial qPCR and 5 min at 95 °C followed by 40 cycles of 95 °C for 30 s, 53 °C for 30 s and 72 °C for 30 s for fungal qPCR^57^. Reactions were followed by a melting curve increasing 1 °C every 30 s from 60 °C to 99 °C. For each community, five biological replicates were amplified. Bacterial copy number was quantified by using 1114f-1275r primers to amplify the 16s rRNA gene from an *Enterobacter* sp. previously isolated from the same soil community as a standard. The amplified product was run on a 1% (w/v) agrose gel and sequenced to confirm the specificity of the amplification. Standard curves were generated using triplicate 10-fold dilutions of the purified amplicon. To create a fungal standard, DNA isolated from representative communities and pure cultures of various soil fungi were amplified with ITS1f-5.8S and visualized on a 2% (w/v) agarose gel. The ITS amplicon from a pure fungal isolate (*Penicillium radicum*), which was closest in size to the mean of the ITS amplicon profile generated by the test communities on the agarose gel, was purified and used to create a standard curve following the same strategy as for the bacterial standard curve.

### Statistical analysis

The R programming language (version 3.2.2) and vegan package were used to generate ordinations of ARISA data using Principal Coordinates Analysis (PCoA)^58^. Dissimilarity matrices were generated using the Bray-Curtis coefficient. No additional transformations were applied as the data were already normalized by the algorithms used in the binning strategy. Statistical analysis of community structure was performed using two-way crossed ANOSIMs (with replicates) in Primer (version 6.0).

Species indicator tests were performed using the indicspecies package in the R programming language to identify OTUs that were over represented in each treatment and in combinations of treatment groups (i.e. all bulk soils and all rhizosphere)^59^,^60^. Factorial ANOVAs and Tukey’s Honest significant difference (HSD) test were performed using custom spreadsheets in Microsoft Excel.

## Additional Information

**How to cite this article**: Wood, J. L. *et al.* Microbial community dynamics in the rhizosphere of a cadmium hyper-accumulator. *Sci. Rep.* **6**, 36067; doi: 10.1038/srep36067 (2016).

**Publisher’s note**: Springer Nature remains neutral with regard to jurisdictional claims in published maps and institutional affiliations.

## Supplementary Material

Supplementary Information

## Figures and Tables

**Table 1:** Global R values for two-way crossed ANOSIMs of fungal and bacterial ARISA data.

Community	Week	Global R test	
		Rhizosphere_pa_	Cd dose
Bacterial	1	0.48*	0.22*
Bacterial	8	0.45*	0.60*
Fungal	1	0.37*	0.15*
Fungal	8	0.28*	−0.04

*Denotes significant (p < 0.05) R values.

**Table 2:** Community structure parameters describing fungal communities at week 8.

	Cadmium dose rate (mg Cd kg soil^−1^)					
	Bulk soil communities			Rhizosphere communities		
	0	20	100	0	20	100
Shannon-Wiener index	1.90	1.63	1.87	1.73	1.83	1.68
Pielous species evenness	0.57	0.51	0.56	0.56	0.57	0.53
Average OTU count	28^a^	24.4^ab^	28^a^	21.6^b^	24.8^ab^	23.4^ab^

For each parameter, values with different letters in superscript are significantly different (Tukey’s HSD test).

**Table 3:** Community structure parameters describing bacterial communities at week 8.

	Cadmium dose rate (mg Cd kg soil^−1^)					
	Bulk soil communities			Rhizosphere communities		
	0	20	100	0	20	100
Shannon-Wiener index	3.85^a^	3.43^b^	3.28^b^	3.77^a^	3.38^b^	3.18^b^
Pielous species evenness	0.92^a^	0.88^bc^	0.85^b^	0.91^ac^	0.86^b^	0.85^b^
Average OTU count	64.8^a^	50.0^b^	46.8^b^	62.8^a^	51.0^b^	42.6^b^

For each parameter, values with different letters in superscript are significantly different (Tukey’s HSD test). Values are the reported as mean of n = 5 biological replicates ± SE.
