# Supplementary material for: Microbial community dynamics in the rhizosphere of a cadmium hyper-accumulator
Source: Sci Rep. 2016 Nov 2;6:36067. doi: 10.1038/srep36067 (PMC5090975; doi:10.1038/srep36067)
Supplement: Supplementary Information [file srep36067-s1.doc]

Microbial community dynamics in the rhizosphere of a cadmium hyper-accumulator

Wood J.L, Zhang C., Mathews, E.R., Tang, C., Franks, A.E


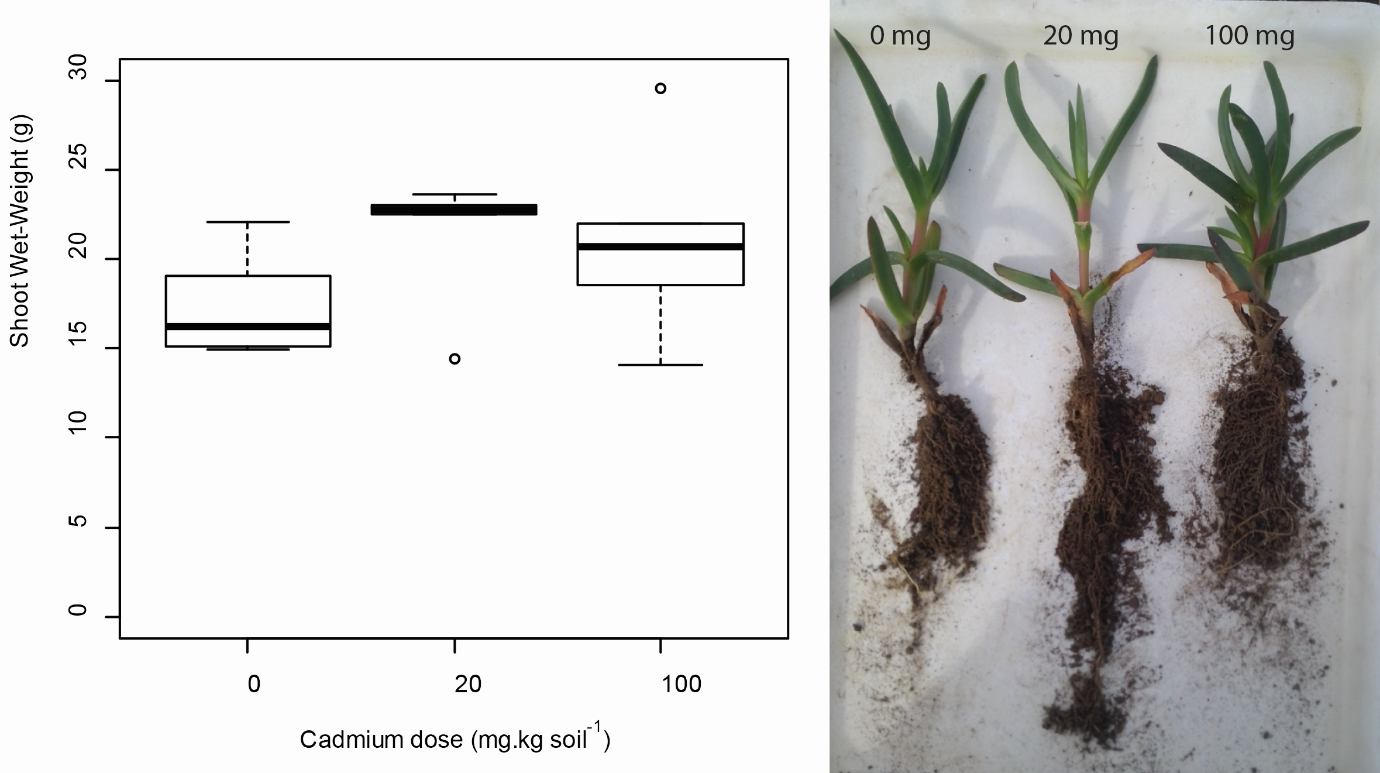


**Figure 1. Effect of cadmium of *C. rossii* growth after eight weeks.** A: box plots of average plant wet-weight for above ground biomass at week eight (n = 5). B: image of representative *C. rossii* plants for each Cd-treatment after eight weeks.


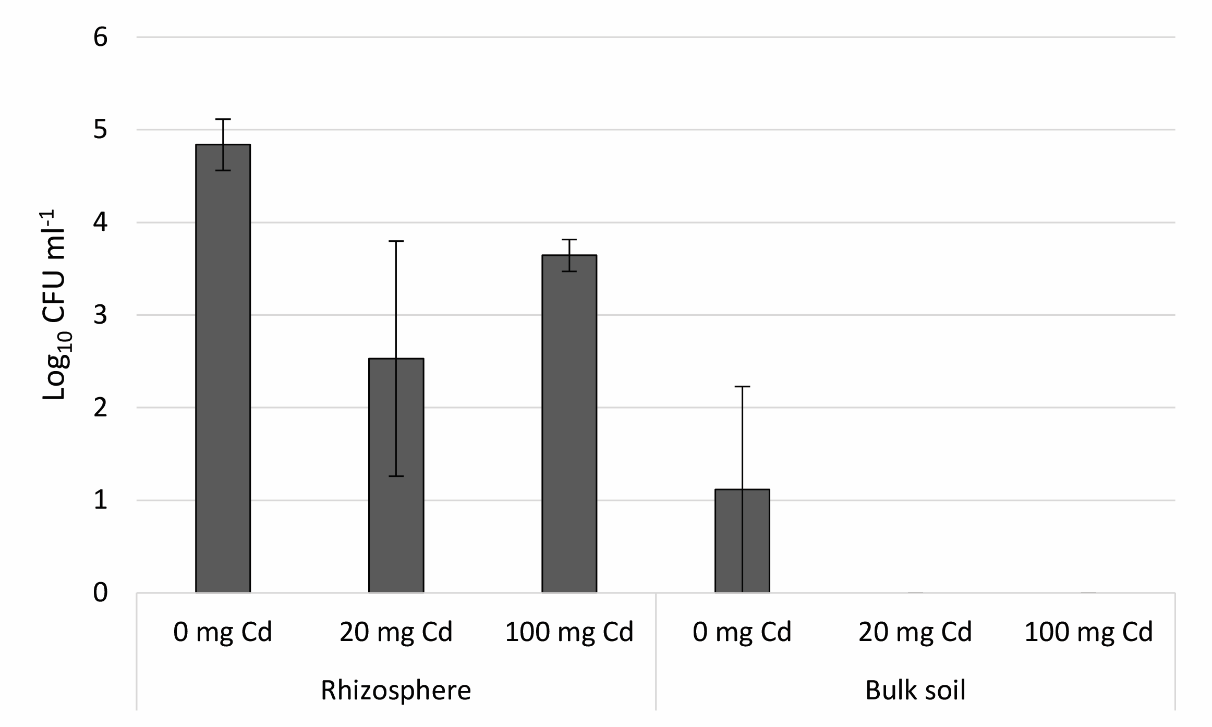


**Figure 2. Pollution induce community tolerance assay for Cd-resistant isolates from week eight soil communities.** Number of CFUs observed to grow on TSA agar containing 2 mM Cd. Error bars = SE, n = 5.
